# Activation of TLR4 by viral glycoproteins: A double-edged sword?

**DOI:** 10.3389/fmicb.2022.1007081

**Published:** 2022-09-29

**Authors:** Emily A. Halajian, Emmanuelle V. LeBlanc, Katrina Gee, Che C. Colpitts

**Affiliations:** Department of Biomedical and Molecular Sciences, Queen’s University, Kingston, ON, Canada

**Keywords:** Toll-like receptor 4, viral glycoproteins, cytokine storm, SARS-CoV-2, dengue

## Abstract

Recognition of viral infection by pattern recognition receptors is paramount for a successful immune response to viral infection. However, an unbalanced proinflammatory response can be detrimental to the host. Recently, multiple studies have identified that the SARS-CoV-2 spike protein activates Toll-like receptor 4 (TLR4), resulting in the induction of proinflammatory cytokine expression. Activation of TLR4 by viral glycoproteins has also been observed in the context of other viral infection models, including respiratory syncytial virus (RSV), dengue virus (DENV) and Ebola virus (EBOV). However, the mechanisms involved in virus-TLR4 interactions have remained unclear. Here, we review viral glycoproteins that act as pathogen-associated molecular patterns to induce an immune response *via* TLR4. We explore the current understanding of the mechanisms underlying how viral glycoproteins are recognized by TLR4 and discuss the contribution of TLR4 activation to viral pathogenesis. We identify contentious findings and research gaps that highlight the importance of understanding viral glycoprotein-mediated TLR4 activation for potential therapeutic approaches.

## Introduction

During the COVID-19 pandemic, a common feature of many severe SARS-CoV-2 cases has been an aggressive inflammatory response characterized by the uncontrollable release of high levels of proinflammatory cytokines, increased activation of immune cells, and harmful hyperinflammation (Mahmudpour et al., 2020; Ragab et al., 2020; Manik and Singh, 2021). This overabundant inflammatory response is termed “cytokine storm,” where a dysfunctional immune response leads to excessive amounts of inflammatory cytokines entering circulation, resulting in organ damage and potentially multi-organ failure and death (Fajgenbaum and June, 2020). Cytokine storms can be triggered from infections with various pathogens, including viruses such as SARS-CoV-2, Ebola virus (EBOV; Younan et al., 2017), dengue virus (DENV; Srikiatkhachorn et al., 2017; Dayarathna et al., 2020), and respiratory syncytial virus (RSV; Rosenberg and Domachowske, 2012).

**Figure 1:**
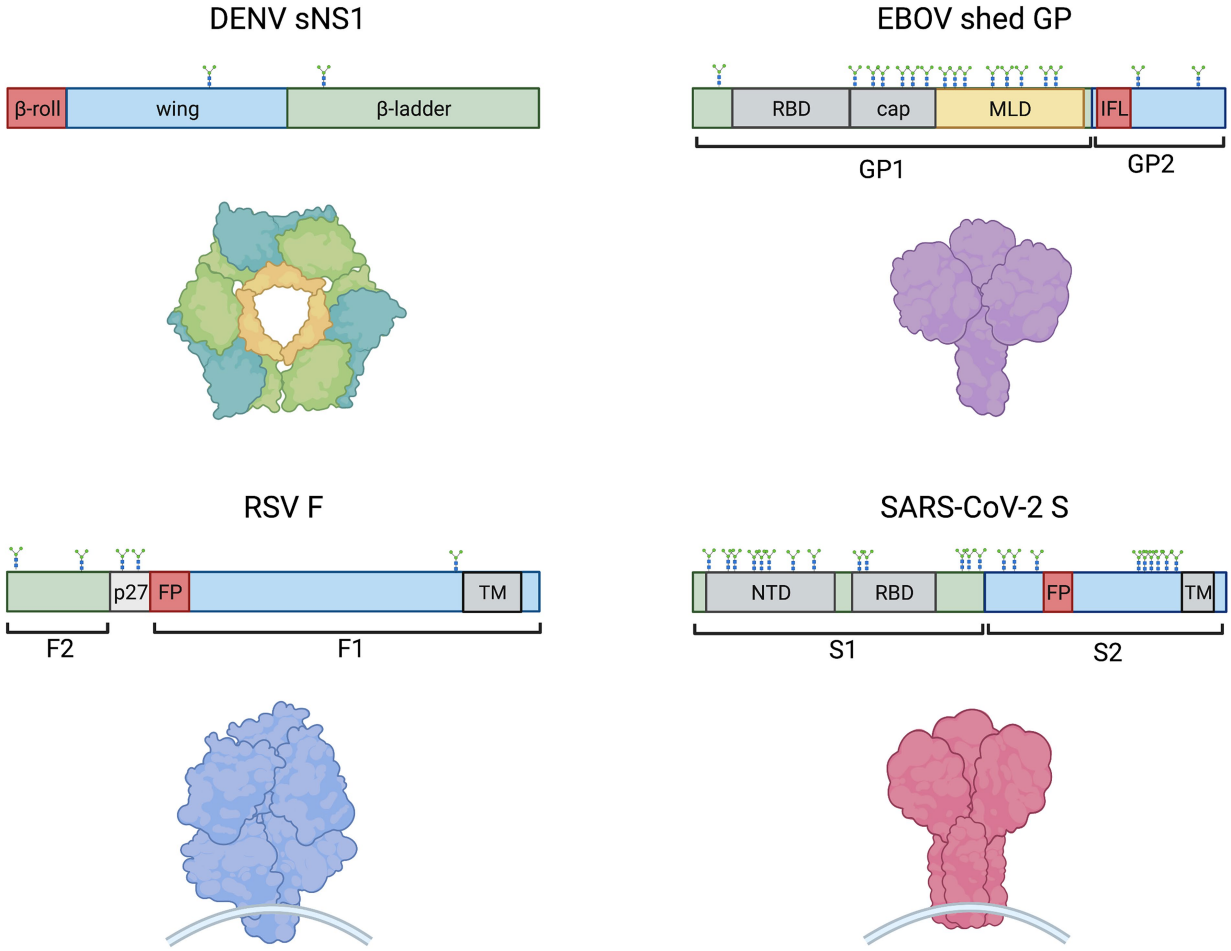
Schematic representations of monomeric DENV sNS1, EBOV shed GP, SARS-CoV-2  S and RSV F (clockwise) showing protein domain structure (above) and models of oligomeric proteins (below). Hydrophobic regions, such as the NS1 β-roll, the GP internal fusion loop (IFL) and fusion peptides (FP) are denoted in red. Other domains are indicated including receptor binding domains (RBD), glycan cap (cap), mucin-like domain (MLD), N-terminal domain (NTD), transmembrane domains (TM), and the RSV peptide 27 (p27). N-linked glycosylation sites are shown. The EBOV MLD shown in yellow also contains ~80 O-linked glycosylation sites (Cook and Lee, 2013).

A shared feature of these cytokine-storm inducing viruses mentioned above, as well as the vesicular stomatitis virus (VSV), is the production of virion-bound or released glycoproteins (Table 1; Figure 1). RSV fusion protein (F), VSV glycoprotein (G), EBOV glycoprotein (GP), and SARS-CoV-2 spike (S) are membrane-associated viral glycoproteins that mediate fusion of viral envelopes with host cell membranes (Olejnik et al., 2018), while DENV non-structural protein 1 (NS1) is a glycoprotein that is secreted from cells in a hexameric soluble form (sNS1) during infection and has exposed hydrophobic domains for membrane interaction (Akey et al., 2014; Figure 1). These proteins share conserved features, such as hydrophobic domains for membrane interactions (NS1 β-roll or RSV F, VSV G, EBOV GP and SARS-CoV-2 S fusion peptides) and glycosylation sites (Figure 1). Notably, each of these viral glycoproteins has been shown to activate Toll-like receptor 4 (TLR4; Olejnik et al., 2018), which plays a role in the induction of cytokine storms (Kaushik et al., 2021; Manik and Singh, 2021), although precisely how these viral proteins activate TLR4 is still unknown. Recently, similarities between systemic inflammation during viral infections and bacterial sepsis (Mahanty and Bray, 2004; Mehedi et al., 2011; Escudero-Pérez et al., 2014; Wolf et al., 2015; Sohn et al., 2020) have been noted. Bacterial sepsis involves an overwhelming and dysregulated host immune response and is characterized by high levels of bacterial lipopolysaccharide (LPS) interacting with TLR4, leading to the overexpression of inflammatory mediators (Kuzmich et al., 2017). Similarly, viral infections leading to overwhelming cytokine responses are often characterized by high levels of viral particles or viral proteins in patients (Mahanty and Bray, 2004; Okumura et al., 2010; Mehedi et al., 2011; Wolf et al., 2015; Guzman et al., 2016; Fajgenbaum and June, 2020; Sohn et al., 2020). Therefore, parallels between LPS-driven bacterial sepsis and virus-induced cytokine storm can be drawn, with a central role for TLR4 in leading to overwhelming systemic inflammation.

**Table 1:** Viral glycoproteins capable of TLR4 activation.

Virus	Viral glycoprotein	References
Dengue virus (DENV)	DENV Nonstructural protein 1 (NS1)	Chao et al., 2019 Coelho et al., 2021 Domínguez-Alemán et al., 2021 Modhiran et al., 2015 2017 Puerta-Guardo et al., 2016 Quirino-Teixeira et al., 2020
Ebola virus (EBOV)	EBOV glycoprotein (GP)	Escudero-Pérez et al., 2014 Iampietro et al., 2017, 2018 Lai et al., 2017 Okumura et al., 2010, 2015 Olejnik et al., 2017 Scherm et al., 2022 Wagstaffe et al., 2020
Respiratory syncytial virus (RSV)	RSV fusion protein (F)	Kurt-Jones et al., 2000 Rallabhandi et al., 2012
Severe acute respiratory syndrome coronavirus 2 (SARS-CoV-2)	SARS-CoV-2 spike protein (S)	Negron et al., 2021 Olajide et al., 2021 Shirato and Kizaki, 2021 Zhao et al., 2021
Vesicular stomatitis virus (VSV)	VSV glycoprotein (G)	Georgel et al., 2007

TLR4 belongs to the Toll-like receptor family of transmembrane proteins that function as pattern recognition receptors (PRRs) recognizing pathogen- and damage-associated molecular patterns (PAMPs; DAMPs) to induce innate immune responses *via* downstream signaling pathways (Lester and Li, 2014; Olejnik et al., 2018). Structurally, TLR4 is comprised of an extracellular leucine rich repeat (LRR) domain, a transmembrane domain, and an intracellular Toll/Interleukin-1 receptor like (TIR) domain with which adaptor proteins TIR domain-containing adaptor protein (TIRAP) and TRIF-related adaptor molecule (TRAM) interact to trigger downstream signaling cascades (Kuzmich et al., 2017). The TLR4 signaling complex consists of cluster of differentiation 14 (CD14), myeloid differentiation factor-2 (MD-2), TLR4, and various adaptor proteins that initiate downstream signaling pathways in a dynamic manner.

**Figure 2:**
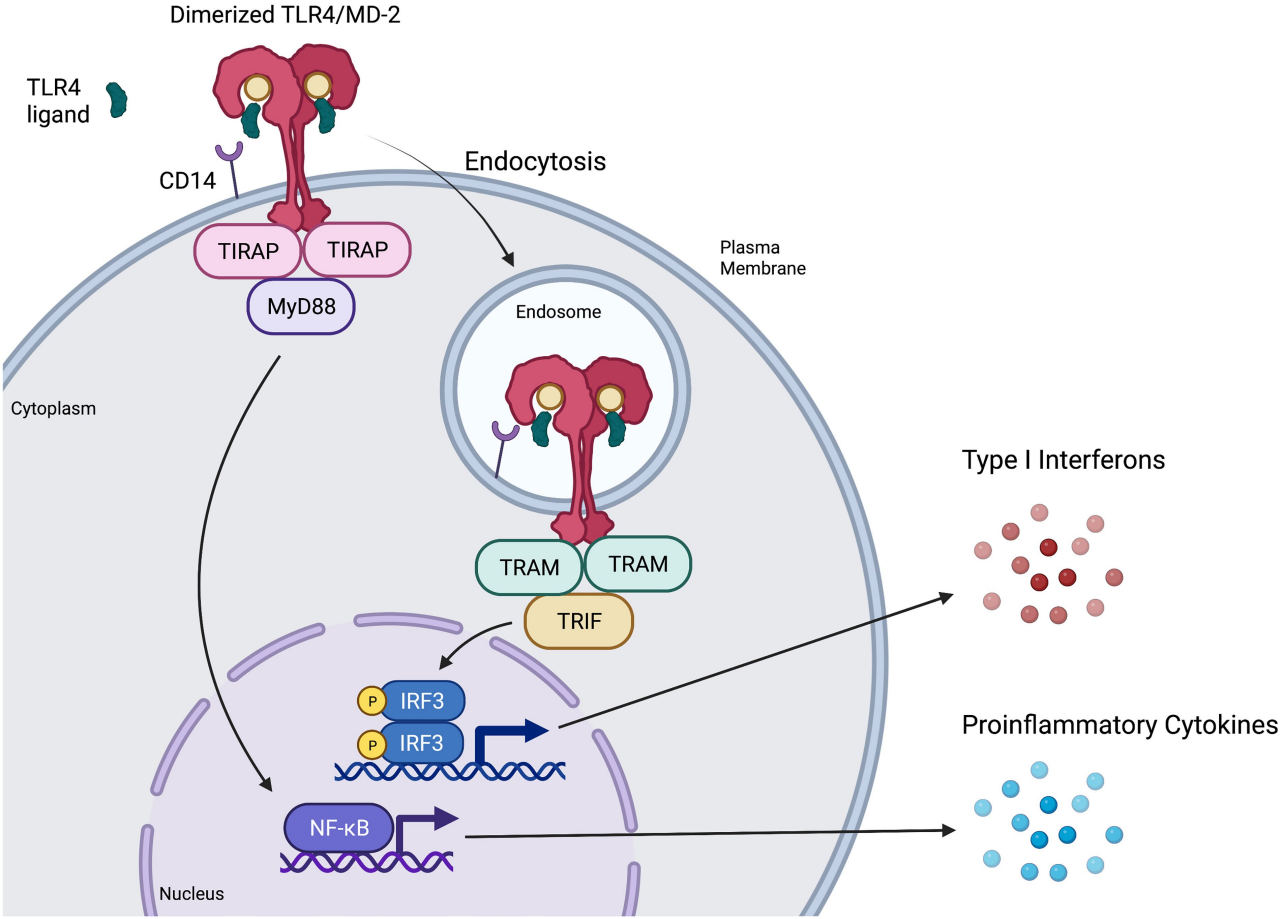
Representation of MyD88-dependent and-independent TLR4 signaling pathways. CD14 presents ligands such as LPS or RSV F protein to individual TLR4/MD-2 complexes, triggering them to dimerize and activate (Park and Lee, 2013; Kuzmich et al., 2017). The TIR domains of the dimerized TLR4/MD-2 complex interacts with TIR domains of the adaptor protein TIRAP (Kuzmich et al., 2017), which then recruits MyD88 to initiate the MyD88-dependent signaling pathway. This pathway results in NF-κB activation and proinflammatory cytokine induction. Once the MyD88-dependent pathway has completed, the dimerized TLR4/MD-2 complex is internalized into an endosome (Kagan et al., 2008; Zanoni et al., 2011), a process that is thought to be at least partially mediated by CD14 and MD-2. Upon internalization, the adaptor protein TRAM interacts with the TIR domains of the dimerized TLR4/MD-2 complex and recruits TRIF, initiating the MyD88-independent (or TRIF-dependent) signaling pathway. The MyD88-independent pathway activates interferon regulatory factor (IRF3) leading to the production of type I interferons (IFN) and activation of interferon-stimulated genes (ISGs).

Classically, CD14 presents monomers of LPS to MD-2 (Kuzmich et al., 2017; Olejnik et al., 2018). Binding of LPS or other ligands to the deep hydrophobic pocket of MD-2 causes TLR4/MD-2 to dimerize and activates the TLR4 signaling complex at the plasma membrane (Park and Lee, 2013; Kuzmich et al., 2017; Olejnik et al., 2018), resulting in recruitment of intracellular adaptor protein TIRAP to then recruit MyD88 (Park et al., 2009). The resulting MyD88-dependent downstream signaling cascade ultimately results in early activation of nuclear factor kappa B (NF-κB) and proinflammatory cytokine secretion (Akira and Takeda, 2004; Figure 2) consistent with cytokine storm. MyD88-dependent signaling activates proinflammatory cytokines such as interleukin (IL)-6, tumour necrosis factor alpha (TNFα), chemokine (C-X-C motif) ligand 1 (CXCL1), IL-1α, IL-1β, and IL-12 (Yamamoto et al., 2003; Björkbacka et al., 2004; Meissner et al., 2013). Experiments in MyD88-deficient cells have shown that lack of MyD88 involves decreased or abolished inflammatory mediator production in response to LPS stimulation (Kawai and Akira, 2007; Yamamoto and Takeda, 2010), although interferon regulatory factor 3 (IRF3) activation and interferon (IFN)-induced gene expression remain unaffected (Kawai et al., 2001; Hoshino et al., 2002).

TLR4 internalization *via* endocytosis is associated with the initiation of MyD88-independent signaling (Kagan et al., 2008; Tanimura et al., 2008; Zanoni et al., 2011; Goulopoulou et al., 2016; Ciesielska et al., 2021). CD14 is thought to have a major role both in regulating the endocytosis of TLR4 (Zanoni et al., 2011; Tan et al., 2015; Marongiu et al., 2019) and in activating the MyD88-independent pathway (Jiang et al., 2005). MD-2 has also shown a potential role in promoting TLR4 transport to the endosome, as well as promoting ligand transport to the TLR4 receptor (Tan et al., 2015). Adaptor protein TRAM is thought to aid in regulating endocytosis of the TLR4 signaling complex (Zanoni et al., 2011). Furthermore, TRAM recruits TIR-domain-containing adapter-inducing IFN-β (TRIF; Tanimura et al., 2008) to induce MyD88-independent signaling (Figure 2), leading to late-wave activation of NF-κB and the induction of some antiviral cytokines and chemokines such as CXCL10 (Yamamoto et al., 2002; Björkbacka et al., 2004). Importantly, MyD88-independent signaling also leads to the activation of IRF3, resulting in the production of type I IFN (Akira and Takeda, 2004; Kagan et al., 2008), induction of IFN-stimulated genes (Kawai et al., 2001) and an antiviral state (O’Neill and Bowie, 2007).

TLR4 signaling is dynamic, with activation of MyD88-dependent and MyD88-independent pathways leading to inflammatory or antiviral responses, respectively (Lester and Li, 2014). MyD88-independent signaling leads to delayed NF-κB activation, IRF3 activation, IFN induction and a beneficial antiviral response. Consistently, studies on MyD88-deficient mice finding reduced proinflammatory cytokine induction but increased activation of IRF3 and IFN-β (Kawai et al., 2001; Hoshino et al., 2002; Yamamoto et al., 2003; Takeda and Akira, 2004). In contrast, triggering of the MyD88-dependent pathway leads to early NF-κB activation and induction of proinflammatory cytokines such as TNFα and IL-6 (Kagan and Medzhitov, 2006), the overproduction of which can have a detrimental effect on the host (Molteni et al., 2016). In the context of bacterial sepsis or viral infection, it is possible that high levels of circulating LPS or viral proteins could continually engage TLR4 on the cell surface, activating MyD88-dependent signaling, while the rate-limiting step of endocytosis may lessen the extent of MyD88-independent signaling in comparison, shifting the balance towards a proinflammatory response consistent with cytokine storm.

While the precise mechanisms explaining how viral glycoproteins may interact with and activate TLR4 and its downstream signaling cascades are still unclear, several potential mechanisms have been proposed. There is some evidence to suggest that physical and hydrophobic interactions occur between these viral glycoproteins and the TLR4 signaling complex, likely through the hydrophobic pocket of MD-2 and hydrophobic fusion peptides or other domains on viral protein surfaces. It has also been proposed that glycosylation may play a role, as all of the viral proteins thought to activate TLR4 are glycosylated. Finally, there is the possibility that a particular oligomeric state of the viral proteins enables interactions with the TLR4 signaling complex. For example, DENV NS1 occurs in both dimeric or hexameric forms during infection, while viral fusion proteins such as EBOV GP, SARS-CoV-2 S, and RSV F are class I fusion proteins that are homotrimers and require proteolytic activation for fusion, which could potentially result in the release of shed fragments of these proteins. Furthermore, the downstream signaling pathways that are induced by specific viral protein-TLR4 interactions still remain to be elucidated. Further understanding of these mechanisms is necessary to determine whether the interaction of viral glycoproteins with TLR4 leads predominantly to an antiviral response or to a proinflammatory cytokine response, or both.

In this review, we summarize the current understanding of TLR4-viral protein interactions, emphasizing the similarities and differences between different viral glycoproteins, including SARS-CoV-2 S. We highlight future directions for investigation and potential therapeutic approaches, which could be employed to ameliorate cytokine storms induced by viruses such as SARS-CoV-2, DENV, EBOV and RSV.

## Current understanding of mechanisms of TLR4 activation by viral glycoproteins

Several viral glycoproteins have been shown to activate TLR4 (Table 1), with experimental evidence for viral glycoprotein-TLR4 interactions ranging from physical interactions between glycoproteins and the TLR4 signaling complex to the induction of downstream signaling pathways and the release of proinflammatory cytokines. In several cases, expression of these cytokines following activation by viral glycoproteins is blocked by TLR4 inhibitors or abrogated in TLR4 deficient models, consistent with a specific role for TLR4. However, the mechanisms underlying TLR4 activation by these proteins is still poorly understood. It should be noted that influenza virus infection has also been associated with TLR4 activation, but this has been attributed to the production of DAMPs such as high mobility group box 1 (HMGB1) from infected cells, rather than a viral glycoprotein (Shirey et al., 2021). While it is possible that DAMPs also contribute to TLR4 activation in the context of other viral infections, we have focused this review on the current literature implicating a specific role for viral glycoproteins in activation of TLR4.

### Physical interaction with TLR4

Due to the role of DENV NS1 in contributing to vascular leakage in dengue hemorrhagic fever/dengue shock syndrome, researchers examined NS1-TLR4 interactions on platelets. Chao et al. (2019) observed decreased levels of NS1 binding to platelets in the presence of anti-TLR4 antibodies, or to platelets from TLR4 knockout mice, indicating that DENV NS1 binds to platelets through TLR4. The same study also compared the binding of NS1 to TLR4 and to TLR2 using enzyme-linked immunosorbent assays and concluded that NS1 had a stronger affinity for TLR4. Quirino-Teixeira et al. (2020) demonstrated that NS1 competed with LPS for binding to human platelets. While it is unlikely that platelets play a role in cytokine storm induction, these studies demonstrated an interaction between NS1 and TLR4. Consistently, studies demonstrated that NS1 and TLR4 colocalized on the surface of peripheral blood mononuclear cells (PBMCs; Modhiran et al., 2015). Similarly, flow cytometry and confocal microscopy approaches showed that EBOV GP binds to TLR4 on T cells (Iampietro et al., 2017), macrophages, and dendritic cells (Escudero-Pérez et al., 2014). Both EBOV GP and SARS-CoV-2 S were observed to interact directly with transfected TLR4 in co-immunoprecipitation studies in HEK 293 T cells (Okumura et al., 2010; Iampietro et al., 2017; Negron et al., 2021). For SARS-CoV-2, it was shown that the S1 subunit (containing the receptor binding domain) but not the S2 subunit (containing the hydrophobic fusion machinery; Figure 1) of the S protein interacted with TLR4 (Negron et al., 2021). Furthermore, the LRR but not TIR domain of TLR4 was required, indicating that the spike S1 subunit binds to the extracellular domain of TLR4. The interaction between TLR4 and the recombinant SARS-CoV-2 spike timer has also been confirmed by surface plasmon resonance (SPR; Zhao et al., 2021). Notably, in the same study, proinflammatory cytokine induction was observed in response to the spike trimer but not to the receptor binding domain (RBD) nor the N-terminal domain (NTD) alone. Based on computational modeling, the authors proposed that TLR4 may interact with a conformational concave constructed by RBD and NTD, both of which are found within the S1 subunit of spike (Figure 1).

In the context of RSV, the purified fusion (F) protein has not yet been shown to directly interact with TLR4, but was observed to co-immunoprecipitate with recombinant MD-2 of the TLR4 signaling complex (Rallabhandi et al., 2012). To identify which portion of the F protein mediated this interaction, the authors made four overlapping polypeptides spanning the length of F1 and observed that only the N-terminal peptide of F1, which contains the hydrophobic fusion peptide (Figure 1), was pulled down in an MD-2/TLR4-dependent manner.

It is still unclear how these viral glycoproteins are recognized by the TLR4 signaling complex. Given that the canonical ligand LPS binds to a hydrophobic pocket of MD-2 to induce TLR4 activation (Park et al., 2009), it is possible that hydrophobic regions of viral proteins may mimic this effect. Indeed, the glycoproteins of VSV, RSV, EBOV and SARS-CoV-2 mediate fusion with host cell membranes using a hydrophobic fusion peptide, and DENV NS1 contains exposed hydrophobic domains. One possibility, therefore, is that viral glycoproteins may mediate TLR4 activation through hydrophobic interactions.

### A potential role for hydrophobic interactions

It was previously shown that NS1 competes with LPS for binding to platelets (Quirino-Teixeira et al., 2020), suggesting that these two ligands may bind TLR4 in a similar manner. Consequently, the TLR4 inhibitor LPS-RS, a potent LPS antagonist which competes with LPS for binding to MD-2 of the TLR4/MD-2 complex (Tam et al., 2021), blocks DENV NS1-induced activation of platelets and murine RAW 264.7 macrophages *in vitro* (Chao et al., 2019; Coelho et al., 2021) and downstream cytokine induction and immune cell activation (Modhiran et al., 2015, 2017). Similarly, LPS-RS prevented EBOV GP-induced NF-κB activation (Olejnik et al., 2017) and reduced GP-induced cytokine production and immune cell recruitment (Lai et al., 2017).

In addition to LPS-RS, Eritoran (a lipid A analog also called E5564), which binds the deep hydrophobic pocket of MD-2 (Kim et al., 2007), inhibited RSV F-mediated TLR4 activation (Rallabhandi et al., 2012), again suggesting a shared binding site on MD-2. The N-terminal fragment peptide of F1, which contains the hydrophobic fusion peptide, reduced LPS-induced NF-κB transcriptional activity, further supporting that it requires the same binding site of MD-2 (Rallabhandi et al., 2012). Similarly, a recent study identified the hydrophobic fusion loop of EBOV GP as essential for TLR4/MD-2 signaling, with docking analysis predicting that the hydrophobic fusion loop binds into the MD-2 pocket (Scherm et al., 2022). Activation of TLR4 by exposed hydrophobic fusion peptides suggests that TLR4 may recognize post-fusion (rather than pre-fusion) conformations of viral glycoproteins, although further experiments are required to explicitly test this possibility.

Consistent with these findings, induction of TNFα expression in THP-1-derived macrophages by SARS-CoV-2 S was also suppressed by LPS-RS (Shirato and Kizaki, 2021). While molecular docking studies have provided some insight, structural analyses to understand the molecular basis for the recognition of these viral proteins by the TLR4 signaling complex are lacking. Notably, molecular binding models of the SARS-CoV-2 S trimer to the TLR4/MD-2 dimer have been proposed (Zhao et al., 2021), showing the protein surface representations and glycans. TLR4 and MD-2 glycosylation are thought to play an important role in the LPS recognition and signal transduction for cell activation (da Silva Correia and Ulevitch, 2002). However, the roles of glycans on the viral glycoproteins in TLR4 activation are still under investigation.

### A potential role for glycosylation

Considering that EBOV GP, DENV NS1, RSV F, VSV G, and SARS-CoV-2 S proteins are glycosylated, and that the core saccharides of LPS may modulate endotoxin activity (Cochet and Peri, 2017), it has been hypothesized that glycosylation of the viral proteins could have a role in TLR4-viral glycoprotein interactions. N-linked glycans on cellular proteins are highly processed into hybrid and complex-type glycan structures, whereas those on viral glycoproteins are often under-processed, resulting in oligomannose-type glycans not typically found on cellular proteins (Watanabe et al., 2019). It is possible that aberrant glycosylation patterns could activate TLR4. To date, however, only glycosylation of EBOV GP has been demonstrated to have a role in TLR4 activation, although these findings remain controversial. By treating EBOV shed GP with a combination of deglycosylases, researchers observed a reduction in TLR4-dependent TNFα secretion by both dendritic cells and macrophages, suggesting that the glycosylation pattern of EBOV shed GP is important for the recognition by TLR4 on immune cells (Escudero-Pérez et al., 2014). Contrarily, Scherm et al. (2022) designed EBOV GP mutants for individual N-glycosylation sites identified by glycosylation prediction software and found that all of these mutants could still activate TLR4 similarly to wild-type EBOV GP. While global deglycosylation of shed EBOV GP by glycosidase treatment significantly reduced TLR4 activation, consistent with the findings of Escudero-Pérez et al. (2014), Scherm et al. (2022) attributed this phenotype to instability or degradation of GP, rather than a specific role for N-linked or O-linked glycans on GP.

EBOV GP has a mucin-like domain that is rich in N- and O-glycans. Co-immunoprecipitation studies revealed that a mucin domain deletion mutant of GP retained the ability to interact with TLR4 (Okumura et al., 2010), suggesting that interaction with TLR4 is not dependent on glycosylation. Consistently, deglycosylated GP was still capable of binding both TLR4 and MD-2 (Scherm et al., 2022). However, EBOV virus-like particles (VLPs) with the mucin domain of GP deleted did not activate NF-κB reporter activity (Okumura et al., 2010), which could reflect instability or degradation of GP, consistent with the findings of Scherm et al. (2022). While the current evidence suggests that glycosylation does not play a direct role in activation of TLR4 by EBOV GP, future studies are needed to evaluate the role of glycans for other viral glycoproteins that activate TLR4.

Lectins, ubiquitously expressed carbohydrate-binding proteins, have been reported to interact with TLRs and have immunomodulatory properties. For example, mannan-binding lectin (MBL) can bind to TLR4 and suppress LPS-induced proinflammatory cytokine production (Wang et al., 2011), although multiple other lectins are reported to act as potent TLR4 agonists (Ricci-Azevedo et al., 2017). It has been proposed that lectin binding to N-linked glycans on TLRs can directly or indirectly activate receptors and induce cell signaling (Ricci-Azevedo et al., 2017). It is interesting to note that several lectins described to activate TLR4 (Park et al., 2010; Unitt and Hornigold, 2011) have specificity for terminal galactose or N-acetylgalactosamine. The spike protein of certain coronaviruses, including SARS-CoV-2, possess a “galectin-fold” with structural homology to human galectin-3, which may have potential relevance for monocyte activation by SARS-CoV-2 (Schroeder and Bieneman, 2022). However, further experiments are required to assess the relevance of the galectin fold for activation of TLR4 by SARS-CoV-2 spike.

### The role of secreted or shed viral glycoproteins

Viral glycoproteins such as EBOV GP and DENV NS1 occur in multiple forms (e.g., shed, secreted, cell-associated or virion-associated) during infection. The question of whether the glycoproteins must occur in a specific form such as shed or secreted, virion- or non-virion associated, to interact with TLR4 remains to be fully addressed. Unlike other TLR4-activating viral glycoproteins, DENV NS1 is a non-structural viral protein that can be secreted as a hexamer formed by a trimer of stable NS1 dimers from DENV-infected insect or mammalian cells (Modhiran et al., 2017). N-linked glycosylation of sNS1 is required for its stability (Somnuke et al., 2011), allowing sNS1 to circulate at high levels in the blood of dengue patients for the duration of illness (Libraty et al., 2002; Guzman et al., 2016). Interestingly, sNS1 produced from insect and mammalian cells activated immune cells in a TLR4-dependent manner, whereas NS1 derived from *E. coli* did not (Modhiran et al., 2017). *E. coli*-derived NS1 lacks glycosylation and is produced only as a monomer (Modhiran et al., 2017), implicating NS1 glycosylation or quaternary structure in activation of TLR4.

*In vitro* and *in vivo* studies have demonstrated that secreted NS1 plays a crucial role in severe dengue immunopathology (Libraty et al., 2002; Carvalho et al., 2014), with levels of circulating sNS1 correlating with risk of developing dengue hemorrhagic fever, a more severe form of dengue disease. TLR4 recognizes and interacts with sNS1 and is a mediator for some of the key roles associated with sNS1 in DENV pathogenesis, including elicitation of inflammatory cytokine production (Modhiran et al., 2015), increased DENV platelet activation (Quirino-Teixeira et al., 2020), and enhanced DENV attachment to host cells (Coelho et al., 2021), as well as endothelial glycocalyx layer disruption (Puerta-Guardo et al., 2016).

EBOV GP is found on the virion surface as a trimer, but is also present in the form of a non-structural soluble GP (sGP) dimer secreted from infected cells, and as shed GP, which is a truncated form of GP cleaved from the plasma membrane by cellular proteases. Early studies on EBOV compared the authentic virus with VLPs or purified GP. EBOV GP alone or VLPs containing GP, but not VLPs without GP, were found to induce TLR4-mediated NF-κB activation (Okumura et al., 2010; Olejnik et al., 2017) and induce suppressor of cytokine signaling 1 (SOCS1) RNA expression (Okumura et al., 2010), highlighting the role of GP as the trigger for TLR4 activation. Escudero-Pérez et al. (2014) demonstrated that shed GP but not secreted sGP activates dendritic cells and macrophages, resulting in a substantial upregulation of several cytokines in a TLR4-dependent manner. Iampietro et al. (2018) later observed that cell supernatant containing shed GP was sufficient to induce monocyte differentiation, an effect requiring functional TLR4, and ultimately resulting in increased infection and cell death. Recently, Scherm et al. (2022) demonstrated that cleavage by TACE protease is required for activation of TLR4/MD-2 by EBOV GP.

While DENV sNS1 and EBOV shed GP are likely the major forms associated with TLR4 activation, it is unclear if there are analogous counterparts for RSV and SARS-CoV-2 glycoproteins. Although many studies have reported that recombinant RSV F protein mediates TLR4 activation, Marr and Turvey (2012) observed no NF-κB transcriptional activity in HEK293 reporter cells transfected with TLR4, MD-2 and CD14 in response to three strains of authentic RSV. One explanation could be that it is not virion-associated F that triggers TLR4, but rather a soluble secreted or truncated form. While F2 remains covalently attached to membrane-associated F1 through disulfide bonds, there is a small segment, p27, between two furin cleavage sites that must be released, as both cleavage events are required for fusion (González-Reyes et al., 2001). Nevertheless, Lizundia et al. (2008) found that NF-κB activity was induced in response to the A2 strain of RSV but not purified F protein, using the same HEK293 reporter system. Thus, there is still no consensus as to which form of F activates TLR4 in a physiologically relevant context. Notably, a secreted form of the RSV attachment protein G has been described (Roberts et al., 1994). Secreted RSV G acts as an antigen decoy (Bukreyev et al., 2008) and has also been proposed to modulate cellular immunity by interacting with TLRs. One study proposed that secreted soluble G inhibits TLR3/TLR4-mediated ISG activation *via* the TRIF pathway, providing an explanation for the lack of IFN-β induction in dendritic cells in response to RSV (Shingai et al., 2008). However, RSV G may have a role in mediating the proinflammatory response against RSV, as a recent study suggested a role for RSV G in binding to and activating the TLR2/TLR6 heterodimer, with a potential partial role in activating TLR4 also described (Alshaghdali et al., 2021).

Khan et al. (2021) observed that SARS-CoV-2 S1 or S2 peptides induced proinflammatory cytokines in lung epithelial A549 and Calu-3 cells, but transfecting these cells with a plasmid encoding S did not result in induction, suggesting that cell-associated S does not induce proinflammatory cytokines. Since SARS-CoV-2 S1 is sufficient to mediate interaction and activation of TLR4, it has been suggested that circulating S1, which may be released from the viral surface following proteolytic cleavage, could be the trigger for a TLR4-mediated inflammatory response (Negron et al., 2021). While the exact form or epitope of viral glycoproteins required to interact with TLR4 remain under investigation, the current knowledge of the resulting signaling and implications for pathogenesis are explored below.

### Activation of signaling pathways and cytokine production

Upon recognition of specific ligands, the TLR4 signaling complex induces downstream signaling pathways. Activation of these pathways begins with the recruitment of adaptor proteins such as MyD88 or TRIF. The MyD88-dependent pathway results in the activation of NF-κB and the production of proinflammatory cytokines, while the MyD88-independent pathway results in the production of IFNs (Figure 2). Determining which signaling pathway is activated and which proinflammatory cytokines and chemokines are produced from each viral glycoprotein-TLR4 interaction will aid in understanding the mechanisms underlying how each viral glycoprotein activates TLR4 and the role of this interaction in viral pathogenesis.

VSV G was shown to activate the TLR4 signaling pathway in a TRAM-dependent MyD88-independent manner that does not activate NF-κB (Georgel et al., 2007). In response to VSV G, myeloid dendritic cells produced type-I IFN in a CD14- and TLR4-dependent manner. Consequently, TLR4- or CD14-deficient murine macrophages were much more susceptible to VSV infection. In the context of *in vivo* VSV infection, TLR4-mutant mice known to be defective for TLR4 responses had a higher mortality rate than their wildtype counterparts, highlighting the importance of TLR4 in antiviral defense against VSV (Georgel et al., 2007). While no studies to date have investigated the mechanisms underlying TLR4 activation by VSV G, it may be that its ligand for TLR4 is presented only in endosomes (e.g., the hydrophobic fusion peptide exposed in the post-fusion conformation), thus bypassing activation of cell-surface MyD88-dependent TLR4 signaling that would otherwise lead to proinflammatory responses.

In contrast to VSV G, TLR4 activation by other viral glycoproteins appears to lead to a disproportionate proinflammatory response. DENV sNS1 induces cytokine release upon interaction with TLR4 (Modhiran et al., 2017). In one study, cytokine responses to sNS1 were lost in mice lacking TLR4, MyD88, or TRIF, suggesting that DENV NS1 interaction with TLR4 triggers both the MyD88-dependent and-independent signaling pathways (Modhiran et al., 2015). EBOV GP has also been found to activate both the MyD88-dependent and-independent downstream signaling pathways (Iampietro et al., 2017; Olejnik et al., 2017). Reporter assays in TLR4/MD-2 expressing cells revealed that EBOV GP induced the expression of genes driven by both NF-κB and IFN-β promoters (Okumura et al., 2010). Consequently, EBOV GP stimulates increased cytokine (Lai et al., 2017; Wagstaffe et al., 2020) and SOCS1 or SOCS3 expression (Okumura et al., 2010, 2015). Multiple studies have shown that NF-κB activation by EBOV GP is dependent on TLR4 (Okumura et al., 2010; Iampietro et al., 2017; Olejnik et al., 2017). Consequently, blocking TLR4 activity with the anti-TLR4 antibodies or TLR4 inhibitors prevented EBOV GP-induced release of various cytokines (Escudero-Pérez et al., 2014; Lai et al., 2017; Wagstaffe et al., 2020), recruitment of antigen-presenting cells (Lai et al., 2017), cell death (Okumura et al., 2015; Iampietro et al., 2017, 2018), and the differentiation and activation of various immune cell types (Iampietro et al., 2017, 2018; Wagstaffe et al., 2020). Thus, unlike in the context of VSV infection, where TLR4 plays a clearly protective role, dampening signaling mediated by TLR4 in response to EBOV or DENV may be beneficial. While some TLR4 activation may be crucial for the activation of some immune cell types, the proinflammatory response may be disproportionately detrimental.

Interestingly, the *in vivo* role of TLR4 in response to RSV infection remains controversial although, like EBOV GP and DENV NS1, RSV F has been shown to trigger the increased secretion of proinflammatory cytokines *via* TLR4/MD-2/CD14 recognition and signaling (Kurt-Jones et al., 2000). *In vitro* studies have confirmed the importance of both MD-2 and CD14, the same components of the signaling complex involved in LPS-induced TLR4 activation (Kurt-Jones et al., 2000; Rallabhandi et al., 2012). The physiological relevance of the resulting signaling and effect on pathogenesis has been well explored *in vivo*. Inoculation of mice with RSV leads to rapid increase in NF-κB DNA-binding activity in nuclear extracts of lung tissue, which was dependent on alveolar macrophages and required functional TLR4 (Haeberle et al., 2002). Notably, the pioneering study in this area found that TLR4 deficient mice infected intranasally with RSV had a higher viral burden in their lungs, suggesting that TLR4 is important in RSV clearance (Kurt-Jones et al., 2000). However, it was later noted that the mice strain used (C57BL10/ScCr) not only had a TLR4 deletion, but also a defect in the IL-12 receptor. However, Haynes et al. (2001) used C57BL/10ScNCr mice (which are distinct from the C57BL10/ScCr strain in that they carry wild-type IL-12R) to confirm that the lack of TLR4 impaired RSV clearance (Haynes et al., 2001). In contrast, Ehl et al. (2004) leveraged several C57BL/10 and BALB/c mouse strains lacking TLR4, IL-12R or both to conclude that TLR4 had no impact on RSV elimination *in vivo* (Ehl et al., 2004). Thus, the role of TLR4 in RSV infection appears variable, depending on the mouse strain used.

As with RSV F, the response to SARS-CoV-2 S is proinflammatory. Both S1 and S2 peptides have been shown to increase proinflammatory cytokine expression in THP-1 derived macrophages while having no effect on IFN-α, IFN-β or INF-γ induction (Chiok et al., 2021; Khan et al., 2021). Reporter assays in HEK293T cells revealed that, like RSV F, SARS-CoV-2 S stimulates NF-κB transcriptional activity (Negron et al., 2021). SARS-CoV-2 S1-mediated activation of NF-κB and MAPK pathways has now been demonstrated in human PBMCs, murine peritoneal macrophages, and murine microglial cells (Olajide et al., 2021, 2022; Shirato and Kizaki, 2021). Proinflammatory cytokine induction in response to S1, S trimer, S pseudoparticles or authentic virus was decreased in the presence of inhibitors of NF-κB, c-Jun N-terminal kinase (JNK), TLR4, MD-2, or by treatment with anti-CD14 antibody (Shirato and Kizaki, 2021; Zhao et al., 2021). The proinflammatory response to SARS-CoV-2 S was also abrogated in bone marrow derived macrophages from TLR4^−/−^ mice (Zhao et al., 2021) or with siRNA targeting TLR4 (Shirato and Kizaki, 2021; Olajide et al., 2022), further confirming the importance of TLR4 in the induction of proinflammatory cytokines. Nonetheless, further research is needed to understand whether TLR4 activation has a protective or detrimental role in viral infection, and the relative role of TLR4 compared to other PRRs.

## The role of TLR4 and spike in the proinflammatory response to SARS-CoV-2

Many studies identified a role for SARS-CoV-2 S in the activation of TLR4 using recombinant purified S trimer or S1, either produced in house or purchased commercially. Cinquegrani et al. (2022) investigated multiple sources of recombinant SARS-CoV-2 S1, checking each for levels of endotoxin contamination, and observed a varied response to these purified proteins in monocyte-derived macrophages, and ultimately proposed that the activation of macrophages correlated with endotoxin contamination or with lack of glycosylation for S1 produced in *E. coli* as opposed to mammalian cells. However, some studies have confirmed their findings with S pseudotyped lentiviral particles or authentic virus (Chiok et al., 2021; Zhao et al., 2021), indicating that endotoxin contamination is unlikely to be the sole driver for proinflammatory responses. Still, others also report a lack of authentic activation in their model systems. For instance, monocyte-derived dendritic cells, which abundantly express TLR4 and other TLRs, were not activated by authentic SARS-CoV-2 particles, S pseudoparticles or recombinant S protein (van der Donk et al., 2022). While the evidence points towards S1 or S2 as the trigger for a proinflammatory response, and TLR4 or TLR2 as the primary mediator of this response (Khan et al., 2021; Shirato and Kizaki, 2021; Zhao et al., 2021; Frank et al., 2022), another study suggested a role for the SARS-CoV-2 E protein in the production of inflammatory cytokines (Zheng et al., 2021). The interplay between SARS-CoV-2 and TLRs is clearly complex and worthy of further investigation, as it is crucial to understand the immunopathology of COVID-19.

## Potential for therapeutic applications

Due to the role of TLR4 in modulating an immune response, the use of TLR4 agonists as potential vaccine adjuvants for DENV, EBOV and RSV have been proposed and are under investigation (Sunay et al., 2019; Zheng et al., 2020; Kayesh et al., 2021). However, in the context of infection, dampening TLR4 activation may be beneficial. Thus, research into the use of TLR4 inhibitors in the treatment of EBOV infection has been recommended (Denner, 2015). In animal models of sepsis, dampening TLR-induced inflammation led to reduced sepsis progression. Specifically, reducing or blocking MyD88 led to decreased systemic hyperinflammation (Weighardt et al., 2002; Weighardt and Holzmann, 2008). These findings could potentially be applied to virus-induced TLR4 activation, as overwhelming activation of TLR4 by LPS in sepsis may be similar to the induction of cytokine storm by virus glycoprotein-TLR4 interactions (Perrin-Cocon et al., 2017; Sohn et al., 2020).

As reviewed here, *in vitro* studies have demonstrated that TLR4 inhibitors dampen MyD88-dependent signal transduction and the release of inflammatory cytokines in response to EBOV GP, RSV F and SARS-CoV-2 S (Rallabhandi et al., 2012; Iampietro et al., 2017, 2018; Lai et al., 2017; Olejnik et al., 2017; Shirato and Kizaki, 2021; Zhao et al., 2021). Notably, a Japanese pharmaceutical company is participating in collaborative COVID-19 clinical trials to test the efficacy of a TLR4 antagonist, Eritoran (E5564), in reducing cytokine storm (Tsukahara, 2022). Consistently, Eritoran had been found to confer significant benefit in the context of influenza infection in mice by blocking TLR signaling (Shirey et al., 2013). Beyond specific TLR4 inhibitors, dexamethasone was shown to significantly reduce SARS-CoV-2 S1-dependent cytokine induction in human PBMCs (Olajide et al., 2021). Although the mechanism has not been fully elucidated, it was observed that dexamethasone pre-treatment reduced activation of NF-κB and MAPK pathways in PBMCs in response to S1, or in human umbilical vein endothelial cells infected with authentic SARS-CoV-2, further supporting the physiological relevance of this inhibitory activity (Ma et al., 2022). Finally, most recently, SARS-CoV-2 S-binding DNA aptamers that selectively disrupt the SARS-CoV-2 S-TLR4 interaction have been identified (Yang et al., 2022). Aptamer treatment of S trimer or authentic SARS-CoV-2 prevented proinflammatory cytokine production by monocytes and neutrophils, while maintaining responsiveness to LPS stimulation (Yang et al., 2022). The specific anti-inflammatory activity *in vitro* and demonstrated low immunogenicity in mice warrant further investigations into the potential therapeutic utility of these aptamers.

Overall, further *in vitro* and *in vivo* studies will be important to elucidate the molecular mechanisms and better understand the physiological relevance of viral glycoprotein-TLR4 activation. These studies are required to explore the therapeutic potential of targeting TLR4 in the context of viral infection to ameliorate cytokine storm.

## Future directions

Further characterization of the mechanisms underlying TLR4 recognition of viral glycoproteins is critical to understand unbalanced inflammatory responses leading to cytokine storm during viral infection. Structural and biochemical studies may elucidate a common molecular basis for viral glycoprotein-TLR4 complex interaction and activation. Further research to identify specific domains of viral glycoproteins that interact with TLR4, as well as to determine how the viral glycoprotein-TLR4 interaction leads to uncontrolled inflammatory cytokine responses, as opposed to a controlled anti-viral response (Olejnik et al., 2018), will be instrumental in understanding viral disease pathogenesis and identifying targets for therapeutic intervention.

The outcomes of viral glycoprotein-TLR4 interactions are complex. Multiple studies on DENV NS1 and EBOV GP demonstrated that these viral glycoproteins induce differentiation and activation of various immune cells (Iampietro et al., 2017, 2018; Olejnik et al., 2017; Wagstaffe et al., 2020; Coelho et al., 2021), emphasizing a broad role for viral glycoprotein-TLR4 interactions in immune responses. NS1-TLR4 interactions modulate the pathogenesis of DENV through a variety of mechanisms, including lipid raft accumulation for cell attachment (Coelho et al., 2021), disruption of the endothelial glycocalyx layer (Puerta-Guardo et al., 2016), platelet activation (Quirino-Teixeira et al., 2020), endocan (a biomarker for endothelial cell activation) expression (Domínguez-Alemán et al., 2021), thrombocytopenia and hemorrhage (Chao et al., 2019). The NS1-TLR4 interaction has also been implicated in vascular leakage (Modhiran et al., 2015), although further studies are necessary to elucidate the relative importance of TLR4 activation (Glasner et al., 2017). A potential mediator of the signal transduction and immune response mediated by TLR4 is its localization at the plasma membrane or in endosomes. Researchers have raised the possibility of EBOV GP interacting with internal TLR4 as well as surface TLR4 (Iampietro et al., 2017). The localization of TLR4 interaction and activation remains to be characterized for DENV NS1, RSV F, and SARS-CoV-2 S, particularly since recent findings indicate that immune cells can be non-productively infected with SARS-CoV-2 (Junqueira et al., 2022; Sefik et al., 2022).

The role of TLR4 in viral infections could be further explored and informed by population studies of TLR4 polymorphisms. Two TLR4 polymorphisms, D299G and T399I, have been associated with hyporesponsiveness to LPS and increased incidence of bacterial sepsis (Arbour et al., 2000; Lorenz et al., 2002). However, there have been conflicting studies on the association of common human TLR4 polymorphisms (D299G and T399I) with symptomatic RSV infection in children (Tal et al., 2004; Awomoyi et al., 2007; Paulus et al., 2007). Studies in the context of dengue infection have yielded similarly conflicting findings (Djamiatun et al., 2011; Sharma et al., 2016; Posadas-Mondragón et al., 2020), while TLR4 polymorphisms D299G and T399I were associated with COVID-19 severity and cytokine storm (Taha et al., 2021). The conflicting results from these studies may reflect small cohort sizes and differences in study populations. It is also possible that indirect effects of viral infection modulate TLR4 activation. For example, one study evaluating the link between RSV infection, airway inflammation and asthma provided evidence that RSV infection leads to a heightened responsiveness to LPS, mediated by increased TLR4 mRNA production and protein membrane localization in lung epithelial cells after RSV infection (Monick et al., 2003). More generally, viral infections are associated with alterations to the respiratory microbiome and increased colonization of potentially pathogenic bacteria in the upper respiratory tract (Hanada et al., 2018). Such changes in the bacterial community during viral infection could modulate TLR4 activation. Further studies in animal models are necessary to characterize whether viral infection-induced changes to the microbiome alter immune responses and TLR4 activation.

In general, *in vivo* models will be crucial to determine the therapeutic potential of targeting TLR4. For example, the role of TLR4 in RSV infection clearance remains unresolved. As for SARS-CoV-2, S1 was demonstrated to have a role *in vivo*, outside the context of authentic virus infection (Frank et al., 2022). To support the investigation of S1 independent of viral infection it is important to note that a most recent study identified circulating S as a blood biomarker for post-acute sequalae of COVID-19, while detection of SARS-CoV-2 nucleoprotein was lesser, providing some indication that freely circulating S may be a trigger for symptoms (Swank et al., 2022). In the *in vivo* study of Frank et al., it was shown that intra-cisterna magna (ICM) injection of S1 in mice resulted in behavioral changes, including reduced total activity and increased social avoidance, that are consistent with the sickness response to infection. The authors identified that S1 was sufficient to modulate neuroimmune gene expression in several brain regions and increase proinflammatory cytokine secretion in hippocampal tissue, confirming cytokine induction *in vivo*. While the role of specific PRRs remained unaddressed, these findings provide a model for investigating inhibitors that specifically counteract the effects of S1 that are most likely mediated by TLR4 or other PRRs. Further *in vivo* studies of EBOV and DENV infection are warranted to directly test the potential of TLR4 inhibitors as therapeutics for viral infections that result in TLR4 activation and an excessive immune response. Overall, further insight into the interactions of viral glycoproteins with TLR4 is critical for understanding viral pathogenesis and identifying therapeutic interventions to prevent cytokine storm during severe SARS-CoV-2, EBOV, DENV, and RSV infections.

## Author contributions

CC conceptualized the review. CC and KG supervised the review and edited the manuscript. EH and EL conducted the literature review, wrote the first draft, and designed the figures. All authors contributed to the article and approved the submitted version.

## Funding

The laboratories of CC and KG are supported by Discovery Grants from the Natural Sciences and Engineering Research Council of Canada, and the Faculty of Health Sciences Spear-Start grant for Pulmonary and Respiratory Diseases Research (Queen’s University). CC is supported by the Canadian Foundation for Innovation John R. Evans Leaders Fund, the Banting Research Foundation, the J.P. Bickell Foundation for Medical Research, and Queen’s University Research Initiation Grant. EVL is supported by a Vanier Canada Graduate scholarship.

## Conflict of interest

The authors declare that the research was conducted in the absence of any commercial or financial relationships that could be construed as a potential conflict of interest.

## Publisher’s note

All claims expressed in this article are solely those of the authors and do not necessarily represent those of their affiliated organizations, or those of the publisher, the editors and the reviewers. Any product that may be evaluated in this article, or claim that may be made by its manufacturer, is not guaranteed or endorsed by the publisher.
